# Economic significance of biofilms: a multidisciplinary and cross-sectoral challenge

**DOI:** 10.1038/s41522-022-00306-y

**Published:** 2022-05-26

**Authors:** Miguel Cámara, William Green, Cait E. MacPhee, Paulina D. Rakowska, Rasmita Raval, Mark C. Richardson, Joanne Slater-Jefferies, Katerina Steventon, Jeremy S. Webb

**Affiliations:** 1 National Biofilms Innovation Centre, University of Nottingham Biodiscovery Institute, School of Life Sciences, University of Nottingham, Nottingham, UK; 2 National Biofilms Innovation Centre, School of Biological Sciences, University of Southampton, Southampton, UK; 3 National Biofilms Innovation Centre, School of Physics and Astronomy, University of Edinburgh, Edinburgh, UK; 4 National Biofilms Innovation Centre, UK & Open Innovation Hub for Antimicrobial Surfaces, Department of Chemistry, University of Liverpool, Liverpool, UK; 5 National Biofilms Innovation Centre & School of Biological Sciences, University of Southampton, Southampton, UK

**Keywords:** Biofilms, Antimicrobials, Applied microbiology

## Abstract

The increasing awareness of the significance of microbial biofilms across different sectors is continuously revealing new areas of opportunity in the development of innovative technologies in translational research, which can address their detrimental effects, as well as exploit their benefits. Due to the extent of sectors affected by microbial biofilms, capturing their real financial impact has been difficult. This perspective highlights this impact globally, based on figures identified in a recent in-depth market analysis commissioned by the UK’s National Biofilms Innovation Centre (NBIC). The outputs from this analysis and the workshops organised by NBIC on its research strategic themes have revealed the breath of opportunities for translational research in microbial biofilms. However, there are still many outstanding scientific and technological challenges which must be addressed in order to catalyse these opportunities. This perspective discusses some of these challenges.

## Introduction

Microbial biofilms are everywhere as the prevalent life forms for microbes in all systems. As such, they present numerous translational opportunities across sectors. These biofilms exert a major impact on human and animal health, pose food safety challenges, disrupt production from oil and gas wells and contaminate drinking water supplies, but they can be beneficial in other areas such as wastewater treatment processes or increasing the bioavailability of nutrients in the soil and remediating oil spillages^1–5^. The wide impact of biofilms across different sectors represents an area of opportunity for translational research on the prevention, detection, management and engineering of biofilms which are the four main pillars of the UK’s National Biofilms Innovation Centre (NBIC), created in 2017 to catalyse collaborations with industry in the study of biofilms to achieve breakthrough innovation^6^.

**Fig. 1:**
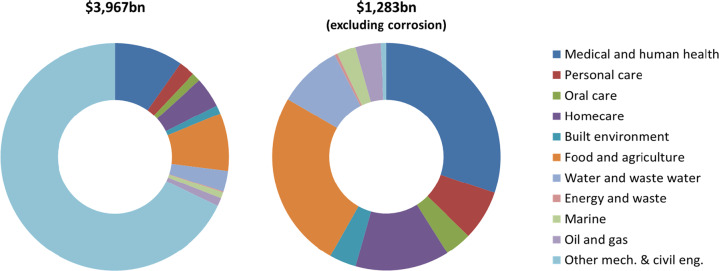
Economic significance of biofilms by sector. Corrosion has been removed from the right chart to expand the viewing of the other sectors.

Capturing the financial implications of biofilms across sectors has been a challenge, as it requires a robust analysis from a diverse range of information sources and the appropriate expertise on the relevant markets. To address this knowledge gap, in this perspective we provide key highlights of the economic impact of biofilms globally in USD ($), based on calculations from a recent market analysis commissioned by NBIC, to provide a better understanding of the real impact of biofilm translational research can have on different sectors. The analysis refers to values in 2019, pre-COVID-19 pandemic, at which point it was estimated that biofilms have an economic significance in excess of $5000bn a year. The breakdown by sectors is provided in Table 1 and illustrated in Fig. 1. The estimated values were taken from a combination of industry reports, academic journal articles and abstracts from specialised market reports. We will also review some of the outstanding scientific and technological challenges that need to be addressed to minimise the negative and maximise the positive financial impact of biofilms.

**Table 1:** Quantification of market sectors engaging with biofilm technologies—summary of economic information^6^.

Sector	Global ($bn)	Comment
Medical and human health		
Wound healing	281	Biofilms form on the surface of wounds and delay healing.
Cystic fibrosis	7.5	The mucus produced in the lungs of cystic fibrosis patients is colonised by pathogens.
Infective endocarditis	16	Biofilms in natural and artificial heart valves result in serious cardiac disease.
Chronic sinusitis	24.4	Chronic sinusitis is often associated with secondary biofilm infections which are difficult to clear.
Ophthalmology	0.759	Surfaces in the eye are prone to biofilm formation.
Human antibiotics	34.2	Bacterial infections are often linked to biofilms and are widely treated with antibiotics.
Central venous catheter bloodstream infection	11.5	Biofilms colonise catheters and can lead to infections.
Catheter-associated urinary tract infection	1	Biofilms colonise catheters and can lead to infections.
Prosthetic cardiac valves and pacemakers	0.22	The surfaces of surgically implanted devices may host biofilms that can only be treated by surgery.
Ventilator-associated pneumonia	2.3	Endotracheal tubes are prone to biofilm infections, which can result in pneumonia and protracted hospital stays.
Breast implants	0.093	The surfaces of breast implants can become infected with biofilms.
Prosthetic joints	7.8	The surfaces of surgically implanted devices may host biofilms that can only be treated by surgery.
Total medical and human health	386.8	
Personal care		
Total personal care	91	Personal care products control biofilms on skin and hair.
Oral care		
Human oral care	47	Tooth scale is a form of biofilm and central to oral health.
Animal oral care	1.85	There is increasing awareness of animal oral health.
Total oral care	48.9	
Homecare		
Homecare	161	Prevention and removal of biofilms contribute to a clean domestic environment.
Textiles	10	Fabrics hostile to biofilms contribute to hygiene.
Total homecare	171	
Built environment		
Cleaning and related hygiene products	41.5	Prevention and removal of biofilms contribute to a clean environment and are essential to some institutions and industry.
Anti-microbial surfaces	7.1	Surfaces hostile to biofilms contribute to hygiene.
Total built environment	48.6	
Food and agriculture		
Crops—microbials	5.3	Biofilm-forming bacteria are used as both biofertilisers and biopesticides.
Crops—antimicrobials	10.4	Prevention of biofilms optimises horticultural output.
Animal husbandry	4.3	Animal health and growth can be promoted by the use of antibiotics to control biofilms in the digestive system.
Food processing	accounted for in ‘crops’	Food safety requires elimination of biofilms.
Preservatives	1.5	Control of biofilms is important to retaining freshness and wholesomeness.
Food packaging	303	Part of the purpose of food packaging is to prevent biofilm growth and control the environment within the packaging.
Total food and agriculture	324	
Water and wastewater		
Water	90.4	Uncontrolled biofilms in water distribution systems present health hazards.
Wastewater treatment	27	Wastewater treatment technologies use biofilms to cleanse water.
Total water and wastewater	117	
Energy and waste		
Anaerobic digestion	2	The composting of putrescible waste by biofilms produces gas and energy.
Landfill gas	3.3	Decomposition of organic matter in landfills by biofilms produces methane which can be captured and used as fuel.
Total energy and waste	5.3	
Marine		
Aquaculture	6	The environmental impact of aquaculture includes the formation of biofilms from feed debris.
Fouling in shipping and other industries	28.2	Biofilms on the hulls of ships increases drag and reduces speed or increases fuel consumption.
Total marine	34.2	
Oil and gas		
Oil spillages	2	Oil in the natural environment is broken down by biofilms that form on its surface.
Corrosion in oil and gas	44	Biofilms produce local chemical environments that are conducive to corrosion that leads to pipeline failure.
Total oil and gas	46	
Mechanical and civil engineering		
Microbial influenced corrosion excluding oil and gas	2676	Microbially influenced corrosion occurs in all industrial sectors.
Corrosion inhibitors	7.52	Inhibition of corrosion requires inhibition of biofilms.
Bioremediation	10.5	
Total mechanical and civil engineering exc. oil and gas	2694	
Grand total	3967

## Healthcare

The impact of biofilms in healthcare has been studied extensively and is one of the areas where a substantial amount of information is available. The US National Institutes of Health reported that over 80% of microbial infections in the body are due to biofilms, many of which are resistant to standard anti-microbial treatments and can require surgery to resolve chronic infection^7^. In addition to the issues caused by antibiotic resistance, the presence of persister cells within biofilms, showing tolerance to antibiotics, represents a challenge for the design of new chemical interventions^8^.

It has been reported that 78.2% of chronic wounds have a biofilm associated with them^9^. In 2017 the global expenditure on wounds in healthcare was $7800bn^10^, of which it is estimated that $281bn corresponds to biofilms.

Biofilms also have a significant impact on the lung and digestive systems of patients with cystic fibrosis^11^. More than 70,000 people worldwide live with cystic fibrosis and around 30,000 of them are in the USA^12^. Biofilms in the CF lung are very resilient to antibiotic treatment and are a major contributor to the overall economic impact of CF, with figures reaching an estimated $7509 m globally^6^ per annum.

Infective endocarditis has a very poor prognosis and although usually caused by bacteria, in a minority of cases it can be caused by fungi^13,14^. The first line of treatment for infective endocarditis is the administration of high doses of antibiotics, but these often fail and surgery is required. There is increasing recognition that biofilms are the major cause of this condition, hampering the ability of the immune system and antibiotics to clear the infection^15,16^. The incidence of infective endocarditis has been on the rise, and a 2013 study reported that the US incidence was 10,000–15,000 cases per year^17^, with a recent estimated annual economic impact of $16bn globally.

Chronic sinusitis or chronic rhinosinusitis (CSR) has been classified as an inflammatory disorder although it is normally associated with secondary bacterial biofilm infections^18^. Topical antibiotics have been used to disperse biofilms, but other attempted treatments include the use surfactants^19^. The estimated direct global cost of CSR was $24.4bn in 2019.

Biofilms present a recurrent issue in catheter-associated infections^20^. For central venous catheters (CVCs) the main route of infection occurs via migration along the surface of the catheter, bypassing the skin and subcutaneous tissue to colonise the intravascular portion of the catheter. Decontamination of CVCs in situ is difficult, likely due to the presence of biofilms^21^, and the incidence of infection is much aggravated by increases in the average length of stay of patients in ICU units in some countries^22^. The annual cost of infections due to CVCs has been estimated at $11.5bn globally. Of equal importance is the economic impact of biofilm formation on catheter-associated urinary tract infections (UTIs), where around 150 million people experience these infections every year, potentially aggravated by the spread of the infection to the kidney. It is estimated that between 15 and 25% hospitalised patients receive urinary catheters, and that catheterisation accounts for around 75% of UTIs in hospitals^23,24^. The estimated global cost of catheter-associated urinary tract infections is estimated to be $1bn annually.

In ophthalmology, biofilm infections can present in the eye and eyelids, but also as a result of contact lenses and artificial lenses introduced during cataract surgery^25^. One of the most prevalent diseases of the eye is keratitis, where the incidence of ulcerative keratitis per 100,000 person every year is approximately nine times higher for contact lens users compared to those who do not use them^26^. The annual global cost of eye conditions associated with biofilms is in the region of $759.3 m.

The insertion of pacemakers and defibrillators under the skin near the collar bone with a wire connection threaded through to a vein into the heart is normally considered a low-risk procedure; however, biofilm infections occur in 1–3% patients within twelve months of implantation^27–29^. This usually requires treatment with antibiotics and, in many cases, the need to replace the device to prevent the development of pneumonia, endocarditis and sepsis^27–29^. Considering that over one million pacemakers are implanted globally per year^30^, and taking into account the expenditure associated with replacing them, the global cost of treating biofilm-related infections in pacemakers and defibrillators is $220 m per year^31,32^.

Endotracheal tubes in patients on ventilators provide a surface prone to bacterial colonisation leading to the formation of biofilms, which can begin to form within 24 h of intubation. Many of these biofilms comprise bacteria found in the oropharyngeal flora, and 50% of these lead to ventilator-associated pneumonia (VAP) upon failure of antibiotics to clear the biofilm. This is aggravated by the fact that patients receiving this treatment are inherently more vulnerable to infections^33–35^. Considering that 35% of the 20,000 ICU beds in the USA are occupied by mechanically ventilated patients, and that the cost of an extra day in ICU is $4000^36–39^, the estimated global impact of biofilms in these patients is $920 m per year.

The surface of prosthetic joints can become infected with biofilms and in many instances antibiotic failure results in surgical replacement. These infections can sometimes appear years after the original surgery and may result in sepsis^40^. There are currently few viable alternatives to surgical treatment^41^. The annual cost of revision surgery due to biofilm-mediated infections is $7849 m globally^42,43^.

## Personal care, oral care and homecare

The personal care sector impacts the quality of life, an ephemeral concept that is difficult to assess economically. Biofilms are associated with and contribute to the cause of skin conditions such as acne, eczema and dandruff, potentially also malodour of intertriginous areas (axilla, vagina), and can inhibit the effectiveness of treatment^44–46^. The reasons consumers purchase personal care products are not consciously associated with the prevention and control of biofilms, but rather linked to perceptions of hygiene, aesthetics and wellbeing. The global personal care market for products is estimated at $339bn (2019), growing by 6.6% annually, with the economic significance of biofilm control estimated at $91bn^47^ of this total.

Oral plaque, a biofilm that forms between teeth and along the gum line, enhances bacterial resistance to the natural host defences and oral cleaning products. Left uncontrolled, it can lead to the demineralisation of teeth and the formation of cavities (i.e. caries) and inflammation of the gums (gingivitis)^48^. Regular removal of dental plaque by cleaning teeth is an important part of healthcare^49^. The dentistry market consists of oral care and hygiene, orthodontics and restorative dentistry, with a global market estimated at $47bn^50^.

A significant part of homecare is concerned with the control or prevention of biofilms for the purposes of health and hygiene but also the sensory environment. The global household cleaning product market is valued at $212bn with 4.97% growth. Growth in the market is driven by rising incomes, increasing urbanisation, less time spent on domestic chores and a consumer shift towards natural, bio-degradable products e.g. surface cleaners, laundry and dishwashing detergents, etc^51^.

## Built environment, mechanical and civil engineering applications

The higher occupant density and increased indoor activity level of some built environments typically increase contact, through both direct person-to-person interactions, or indirect contact via shared surfaces or air. These play a key role in facilitating the spread of microorganisms which can cause disease^52^. The control of biofilms in these environments is at least as important if not more so than in other built environments like the home. This is critical in order to provide microbiological safety for employees, the public (e.g. hospitals and schools) and the provision of safe goods and services (e.g. food that is fit to eat). Products and other resources such as time and energy are used to prevent, clean and detect microbial contamination in water systems, on surfaces, and in the air. The annual global market for cleaning products in these environments is estimated to be $41.5bn^6^. One example in this market is the materials and services for control of *Legionella* in building hot and cold water supplies. *Legionella* grows and multiplies in building water systems and in cooling towers, and aerosolised water droplets containing *Legionella* can be small enough for people to breathe in, potentially causing Legionnaires’ Disease. Facilities management teams must ensure control mechanisms and testing are in place to prevent this from occurring.

There has also been significant innovation in developing surfaces resistant to microbial contamination, and the market for these globally is estimated at $7.1bn per year. The anti-microbial property is often achieved by the application of a coating, or impregnation with active materials including silver or copper. Anti-microbial activity can also arise from the 3D structure of the surface and even photocatalytic effects^53^. The increased emphasis on cleaning surfaces during the Covid-19 pandemic has led to projections that the cleaning market will grow to $68.2bn by 2027^54^.

## Food and agriculture

The world’s 50 million km^2^ of managed soils contain 12.5 billion tons (US) of bacteria comprising an estimated 2.6 × 10^29^ bacterial cells^55^. These bacteria are involved in a wide number of essential processes impacting the production of both plant- and animal-based food. These include the carbon, nitrogen and sulphur cycles as well as the immobilisation of inorganic materials to contribute to land remediation.

The management and control of biofilms throughout the cycle of food production, processing, and distribution, both in agriculture and in the supply chain through to the home, is vital in ensuring efficient food production and food safety. Biofilms are an integral part of the world’s $3700bn of agricultural activity^56^ and are estimated to have a $324bn global impact annually^6^. There is increasing interest in the control and use of biofilms and microbiomes to support agricultural production. For example, the routine use of antibiotics in animal feed was historically widespread, improving welfare by preventing some infections and promoting faster growth. However, due to welfare concerns and the threat of increased antibiotic resistance, antibiotic growth promoters were banned in the EU in 2006, although they remain in use in many developing countries^57^.

In food processing itself the principal method of control of biofilms is through the use of effective but often costly hygiene practices. Where these measures fail, there can be a significant impact on human health through food poisoning, leading not just to human morbidity but also mortality. For example, there were 142 cases of foodborne Listeriosis in the UK in 2019 resulting in 23 deaths and 8 miscarriages or stillbirths^58^. Further resources are devoted to investigating outbreaks of food poisoning so that contaminated products can be recalled, and consumers advised to avoid products. One single example of vegetables contaminated with *Listeria monocytogenes* (the cause of Listeriosis) in the EU was estimated to cost the manufacturer in the region €30 million ($35.1 million). This includes estimated costs for the recall of the product, transport and destruction^59^. In the US, foodborne pathogens are responsible for around 128,000 hospitalisations each year, resulting in an economic impact of $78bn a year^60^.

## Water and wastewater, Energy and waste

Biofilms provide the central mechanism for wastewater treatment. Several process configurations are used for this purpose. In activated sludge processes, aeration and mixing of the waste liquor in the presence of flocs of microorganisms result in the consumption of the organic matter in the wastewater^61^. In attached growth processes, biofilms attached to a supporting surface are exposed to the wastewater. This ensures that there is adequate surface area availability of organisms in the biofilm to treat the water. There are several forms of these so-called attached growth systems; for example, trickling filter systems involve sprinkling wastewater over a vessel filled with inert media covered in biofilm (e.g. stones, coke, ceramics)^62^. Constructed wetland systems are also attached growth system. The biofilms are supported in beds that contain graded growth media (e.g. soil, gravel, sand), which are planted with suitable vegetation. Purification is achieved by means of physical, chemical and biological processes^63^.

The water treatment market was estimated to be worth $262bn globally in 2015, increasing to $313bn per annum by 2018^64^. It is dominated by municipal wastewater treatment (92%) with only 8% for industrial wastewater. Although microbial wastewater treatment has been practiced for generations, it is clear that there is a huge demand for practical and resilient water treatment systems that can serve the demands for sustainable development i.e. reducing capital and operating costs, reducing the burden on the environment, and providing clean water. Biofilms impact all aspects of these systems—the pipeline distribution system, the water treatment system and also the environmental aspects (including energy consumption).

Anaerobic digestion is used industrially for the conversion of waste biomass into renewable energy in batch or continuous processes. Biofilms are key for the conversion of some of the biomass into biogas, which is principally a mixture of methane and carbon dioxide that can be used for power generation. The estimated global impact of the deployment of anaerobic digestion for this purpose is $2bn per year^6^.

## Marine, oil and gas

The major global challenge of our times is climate change. In order to deliver energy efficiency for net-zero carbon emissions, the biofilms community must deliver concerted research and translational effort on a number of fronts, including prevention of biofouling, delivering sustainable anti-biofilm technologies, and combatting microbial-induced corrosion.

Biofouling of shipping vessels, which transport ~80% of global trade^65^, contributes 2% of global carbon dioxide emissions^66^. Biofouling increases the frictional resistance of hulls, increasing fuel consumption by 15–20% for a given speed^67^. Many vessels use sulphur-rich fuels, with the world’s 15 largest ships emitting the equivalent emissions to 760 m cars^68^. Biofouling also increases the density and weight of static marine structures such as offshore renewable energy installations and oil and gas industry infrastructure, increasing the risk of damage or accidents during deployment and decommissioning^69^. Combatting this problem is the anti-fouling marine coatings sector, with an estimated market value increasing to $23bn by 2023^70,71^. Innovation is being driven by sustainability and regulatory changes, and stringent restrictions on new actives. Translation of a new generation of actives or physical approaches to combat biofouling represents an urgent challenge for academia and industry alike.

A serious consequence of marine biofouling is the transfer of invasive species by ships, leading to significant environmental and socioeconomic impacts on fisheries, aquaculture, mariculture and coastal infrastructure^72^. Aquacultural products are estimated to reach 109 m tonnes globally in 2030^73^. However, biofouling of associated infrastructure, with biofilms often harbouring non-native microorganisms, is currently estimated to cost this sector $4-8bn per year^69^.

Much of the infrastructure in the marine and the energy sectors are also vulnerable to microbially influenced corrosion (MIC). Fouled marine surfaces become susceptible to corrosion, and increased risk of damage from heavy waves, impact or vibration. In the oil and gas industry, MIC causes up to 20–40% of serious corrosion cases and up to 70–95% of pipeline leaks^74^. This damage drives the market for corrosion inhibitors, estimated to be worth $7.5bn in 2019^75^. Environmental regulatory changes are phasing out inorganic inhibitors, driving innovation in organic-based alternatives.

## Outstanding scientific challenges to deliver translational impact from biofilms

The economic and societal impacts identified above demonstrate that biofilms are central to some of the most important global challenges, and that the potential for benefit and growth from harnessing and controlling the activities of complex biofilms exists on a considerable scale. However, many scientific challenges remain, and innovation within these areas will rely on an underpinning platform of advances in basic and translational research. A number of the key scientific challenges (by no means intended to be an exhaustive list) are identified below. Further expansion on the themes identified below can be found within a series of academic-industry knowledge exchange workshop reports prepared by NBIC^76–80^.

### Better understanding of the interaction of biofilms with different surfaces

Surfaces and interfaces are pivotal in biofilm prevention strategies across sectors. The key scientific challenges for knowledge-based innovation include understanding how the nature of the surface affects adhesion, early biofilm development, and preferential colonisation of specific species. Equally, understanding the effect of microbes on the surface is important, from conditioning effects to initiation of corrosion events in MIC. Such ‘surface structure/biofilm property’ relationships require nano-to-macro level control and characterisation of the surface, to be combined with tracking microbial behaviour from the single cell to microbiota. As regulatory guidelines become more stringent, sustainable innovation will require developing new classes of surfaces and surface modifications e.g.: bio-inspired systems; increasing diversity and combinations of surface functionalisation to enable precision and broad-based interventions; and development of surfaces with smart anti-microbial delivery systems. Such designed surfaces could also provide anti-microbial resistance (AMR) stewardship. To realise innovation, economically viable upscaling of knowledge-engineered lab materials to production scale is critical.

### Deeper knowledge of the matrixome to design more effective interventions

The matrixome is the assembly of the extracellular polymeric substances (EPS) that contribute to the unique attributes of a biofilm^81^. It poses a physicochemical barrier which presents a challenge to the development of effective anti-microbial interventions. Anti-microbial treatments may be unable to reach deeper areas of the biofilm, e.g. through restrictions in diffusion, or may be chemically modified/inactivated through interactions with components of the EPS^48–50^. Furthermore, our limited fundamental understanding of the physicochemical properties of the EPS in complex polymicrobial communities, such as those found in different industrial/environmental contexts, represents an important hindrance in the discovery of successful interventions^50^. There is a real need to develop novel technologies which enable the study of the biofilm matrix in natural environments, to gain the level of understanding required to design effective chemical and physical biofilm interventions together with the appropriate delivery systems.

### Better rapid early detection systems: e.g. biofilm biomarkers, in situ detection for real-world applications

A key challenge required to drive translation in biofilms is the development of new and biofilm-specific detection technologies that are suitable for in situ, point-of-use contexts, in both industrial and human/medical settings^76,82^. This would be aided through the identification of new, and exploitation of known, biofilm-specific biomarkers, including those for biofilm-associated AMR and resistance gene monitoring. When a biofilm is implicated in an infection, it often cannot be sampled directly without invasive methods, including surgery. This essentially precludes detection until intra-operative access is possible; and further delays then occur while standard microbiological methods such as bacterial culturing on agar plates is performed^82^. Therefore, the capability for rapid, non-destructive and real-time detection in situ has the potential to be transformative for biofilms across a range of sectors. Addressing these challenges will bring exciting new technologies for safer medical devices, better healthcare and early interventions across a range of biofilm challenges including water contamination, microbial-induced corrosion and antibiotic-resistant infections.

### Improved biofilm analysis methods for in-depth understanding: label-free chemical profiling, sensors, AI/modelling

For advances in biofilm detection, diagnosis, and control there is also a critical need for improved biofilm analytical technologies to deepen our understanding of the complexity of biofilm structure and function. From an innovation perspective, it will also be essential to bring forward new and advanced analytical standards, in order to demonstrate the alignment of biofilm products and technologies to new analytical capabilities. Advances are needed in culture-independent technologies coupled with direct integrated ‘omics data analyses that will allow for real-time information about the nature and composition of biofilms. New photonics-based approaches, for example, developments in label-free Raman spectroscopy, together with the potential for miniaturisation and parallelisation for photonics sensor chip technology are areas where interdisciplinary approaches may lead to transformative technologies for biofilm analysis and sensing. AI and machine learning techniques will increasingly be used to exploit large datasets, discover biomarkers and decipher complex relationships within complex microbial consortia.

### Managing biofilms in natural environments

Biofilms in natural ecosystems offer both potential benefits and threats; however, our capability to interrogate complex polymicrobial ecosystems in the natural environment, whether in the gut, in soils or in marine environments, represents a substantial challenge despite significant advances in recent years. Although great strides have been made in determining the composition of complex communities, teasing out the functional roles of individual species to the behaviour of the community as a whole remains, in many cases, a “black box”. Additional complexity comes from the influence of both the host, and the impact of a fluctuating and unpredictable external environment. Advances in low-cost/high-throughput multi-‘omics technologies can be combined with artificial intelligence/ machine learning approaches, mathematical modelling and advanced simulations to build up a picture of these highly complex interactions in situ using a systems approach.

### Improved understanding of the mode of action of antibiotics/antimicrobials and how they interact with biofilms

Most anti-biofilm drug discovery programmes that have made it into clinical trials have been based on prior target knowledge, following a very structured drug optimisation approach^51^. However, the screening of natural products has identified large numbers of compounds with anti-biofilm properties of the yet unknown mechanism of action^52,53^. Many of these have chemical structures not usually found in synthetic-based libraries and hence are likely to exhibit novel modes of action^54^. The integration of artificial intelligence into drug discovery programmes seems a promising strategy to accelerate the identification of targets for these novel chemical entities, although abundant challenges remain^55^.

### The need to improve standards/biofilm models

Despite the clinical and industrial significance of biofilms, few validated or regulatory-approved methods exist with which to evaluate them. This is true not only for laboratory research and testing methodologies but also in situ within real-world settings, for example, to measure the effects of routine treatments against biofilms in engineered water systems, or to measure the outcomes in formal clinical trials of novel anti-biofilm therapeutics. Further compounding factors include that (1) traditional culture leads to a high number of false negatives and is unable to differentiate between planktonic bacteria and biofilms; (2) advanced methods for evaluating biofilms, for example, fluorescent in situ hybridisation (FISH) or immunofluorescence, are specialist and time-intensive; and molecular methods are highly sensitive but cannot differentiate between planktonic and biofilm modes of growth. There is clearly an important research gap, both in basic and applied contexts, in the ability to accurately model, predict and evaluate biofilm activity in real-world settings.

### Lack of biofilm repositories e.g. biobanking

Biobanks and collections of biofilm samples relevant to health and industrial applications are lacking. They are essential for fundamental research on the biology of biofilms but also for the relevant testing and validation of novel interventions. There is a real need to develop biofilm biobanks including polymicrobial communities that retain their physicochemical properties upon storage. These biobanks also require an underpinning digital infrastructure to make them accessible and discoverable. Leveraging existing infrastructure within national capabilities and understanding the requirements of the biofilm community to advance biobanking technology should offer a significant return to the biofilm field.

### Importance of international collaboration and training

As clearly evidenced during the time of a global pandemic, international collaboration is not only critical for the benefit of society but also for the recovery and growth of the economy. Biofilms are central to some of the most urgent global challenges and exert considerable economic impact across industry sectors. To build relevant and value-creating solutions, the national ecosystem and market must be fully understood, within the global context. In February 2020 NBIC, together with the two largest biofilm research centres outside the UK- the US Center for Biofilm Engineering (CBE) and the Singapore Centre for Environmental Life Sciences Engineering (SCELSE), joined efforts with the EU Cooperation in Science and Technology (COST) action group on Anti-MIcrobial Coating Innovations (AMICI), to form a task group which will drive the international development and acceptance of standardised biofilm test methods in healthcare, the built environment, and industrial systems. The goal of this International Biofilms Standards Task Group is to enable informed and consistent decision-making on the international regulation of anti-biofilm products.

International collaboration and interactions within the Higher Education Sector allow graduates and early career researchers to gain new perspectives on their research, enhancing the pedagogical and research outcomes of their work whilst increasing and diversifying their networks globally. International collaboration must bring together partners with complementary strengths and expertise to address industry-led core scientific questions in real-world situations, so as to provide the solutions to the challenges that impact us all.

## Data Availability

Data sharing not applicable to this article as no datasets were generated or analysed during the current study.
